# Acute effect of tendon vibration applied during isometric contraction at two knee angles on maximal knee extension force production

**DOI:** 10.1371/journal.pone.0242324

**Published:** 2020-11-13

**Authors:** Jonathan Harnie, Thomas Cattagni, Christophe Cornu, Peter McNair, Marc Jubeau

**Affiliations:** 1 Laboratoire Motricité, Interactions, Performance EA 4334, Faculty of Sport Sciences, University of Nantes, Nantes, France; 2 Health and Rehabilitation Research Institute, Faculty of Health and Environmental Sciences, Auckland University of Technology, Auckland, New Zealand; University of L'Aquila, ITALY

## Abstract

The aim of the current study was to investigate the effect of a single session of prolonged tendon vibration combined with low submaximal isometric contraction on maximal motor performance. Thirty-two young sedentary adults were assigned into two groups that differed based on the knee angle tested: 90° or 150° (180° = full knee extension). Participants performed two fatigue-inducing exercise protocols: one with three 10 min submaximal (10% of maximal voluntary contraction) knee extensor contractions and patellar tendon vibration (80 Hz) another with submaximal knee extensor contractions only. Before and after each fatigue protocol, maximal voluntary isometric contractions (MVC), voluntary activation level (assessed by the twitch interpolation technique), peak-to-peak amplitude of maximum compound action potentials of vastus medialis and vastus lateralis (assessed by electromyography with the use of electrical nerve stimulation), peak twitch amplitude and peak doublet force were measured. The knee extensor fatigue was significantly (P<0.05) greater in the 90° knee angle group (-20.6% MVC force, P<0.05) than the 150° knee angle group (-8.3% MVC force, P = 0.062). Both peripheral and central alterations could explain the reduction in MVC force at 90° knee angle. However, tendon vibration added to isometric contraction did not exacerbate the reduction in MVC force. These results clearly demonstrate that acute infrapatellar tendon vibration using a commercial apparatus operating at optimal conditions (i.e. contracted and stretched muscle) does not appear to induce knee extensor neuromuscular fatigue in young sedentary subjects.

## Introduction

Over the past two decades, the effects of prolonged tendon vibration on maximal motor performance have been widely investigated. However, the results reported in the literature are contradictory. Some findings showed a decrease in maximal force production for knee extensor (KE) [1–6], plantar flexor [7–9] and dorsiflexor [10] muscles following at least 20 min of tendon/muscle vibration. Prolonged vibration may therefore induce neuromuscular fatigue, defined by a reduction in maximal voluntary contraction (MVC) force [11]. Neuromuscular fatigue is associated with alterations at different levels of the motor pathway from the brain to the skeletal muscle [11,12]. It is classically defined as being proximal (central fatigue) or distal (peripheral fatigue) to the neuromuscular junction, even if neuromuscular fatigue results from a complex interaction between both central and peripheral fatigue [11]. After prolonged vibration, it has been reported that peripheral mechanisms are rarely altered [3,6–8,13–16], suggesting that the decrease in MVC force is mainly explained by neural alterations. In this context, previous studies showed an attenuation of Ia afferent function [1,2,5,7,8], supported by reductions in spinal loop excitability, as evidence by H-reflex measurement [13,14]. However, this reduction in H-reflex excitability is not always associated with a reduction in maximal force production [14,15]. Indeed, recent investigations did not reveal a significant effect of tendon vibration on maximal motor performance for muscle groups crossing the ankle or the knee joints [13–16]. Although experimental parameters such as the vibration frequency used [1,16] have been examined in the literature to maximize fatigue effect following prolonged tendon/muscle vibration, other factors need to be explored such as muscle activity (i.e. contraction) or joint angle (i.e. muscle length) during vibration.

Indeed, it is worth noting that participants in the aforementioned studies were asked to remain relaxed during prolonged tendon vibration. However, muscle contraction implies an increased activity of γ-motor neurons that control the sensitivity of muscle spindle primary endings [17]. In other words, increased levels of γ-motor neuron activation result in more sensitive muscle spindle primary endings. Consequently, a higher discharge rate of spindle muscle endings is observed when tendon vibration is applied during submaximal isometric muscle contraction compared to muscle discharge at rest [18]. Therefore, submaximal muscle contraction performed during prolonged tendon vibration may strengthen the attenuation of Ia afferent function and cause a higher alteration in maximal muscle force production. Whereas some authors used prolonged tendon vibration during submaximal KE muscles contraction for optimizing chronic neuromuscular adaptations [19–22], the acute effects of a single session combining KE muscles contraction and vibration has never been investigated.

Furthermore, the question of the effect of joint angle on neuromuscular fatigue while prolonged tendon vibration is applied remains to be considered. Considering the knee joint model, a change in knee angle from extended to flexed leg causes an elongation of KE muscles [23,24]. It is well known that increasing muscle length implies an enhanced Ia, Ib and secondary afferent sensitivity [25–27]. Furthermore, it has been found that the Ia afferent discharge rate associated with vibration is higher at long muscle lengths than at short muscle lengths [18]. Prolonged KE contraction with tendon vibration at a long muscle length (i.e. flexed knee) should therefore induce more neuromuscular fatigue than at a short muscle length (i.e. extended leg).

The purpose of this study was therefore to assess the effect of a fatiguing exercise including the application of tendon vibration during submaximal isometric KE contraction at two knee angles (i.e. 90° and 150°; 180° = full leg extension) on the maximal motor performance of KE muscles. Knee angles of 90° and 150° were chosen because they correspond to long (stretched) KE muscle length and short (slacked) KE muscle length, respectively [28]. We hypothesized that prolonged weak muscle contraction induced a greater neuromuscular fatigue with concomitant tendon vibration than without vibration. Since tendon vibration especially alters Ia afferent function, we also hypothesized that the neuromuscular fatigue induced by tendon vibration during the submaximal contraction may primarily result from central fatigue. In addition, since the effects of vibration are more effective at long muscle lengths rather than at short muscle lengths, we assumed that the neuromuscular fatigue should be greater at a 90° knee angle compared to a 150° knee angle.

## Methods

### Participants

Thirty-two young adults (25 males, 7 females, 22.1 ± 3.2 years, 174.7 ± 8.2 cm, 67.5 ± 10.3 kg, mean ± standard deviation) with no history of neurological and/or musculoskeletal disorders participated in the experiment. Only sedentary individuals were included, i.e. individual who had less than 2 hours of moderate-to-intensive physical activity per week within the 6 months preceding the experimental session. Selected individuals were volunteers and gave their written consent prior to participation in the investigation after being informed about the nature, aims, possible risks and discomfort associated with the study. They were asked not to engage in any strenuous locomotor activity for at least 48 h before the experimental sessions. The protocol of the current investigation was approved by the ethics committee of Nantes and was in conformity with the Declaration of Helsinki (last modification, 2013).

### Design and procedures

Participants were randomly assigned into two different groups, based on the knee angle tested: i) group^90°^ (performing neuromuscular assessment and fatiguing exercise at 90° knee angle); ii) group^150°^ (performing neuromuscular assessment and fatiguing exercise at 150° knee angle). All tests were carried out on the right leg. Each participant completed two experimental sessions: one in which fatiguing exercise included tendon vibration and submaximal KE contraction (10% MVC); and another in which fatiguing exercise only included submaximal KE contraction at 10% MVC (control session, i.e without vibration). These two experimental sessions were randomly administered to the participants at the same time of day and were interspaced by an interval of 4 weeks.

**Fig 1:**
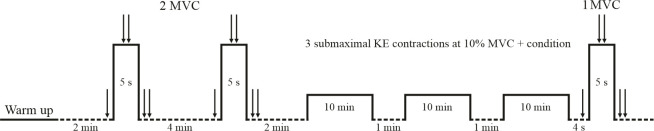
Illustration of the experimental protocol. MVC: maximal voluntary contraction; 1 arrow: single electrical stimulus; 2 arrows: doublet stimulation (10 ms between 2 electrical stimuli); condition: with vibration or without vibration; dotted line: rest period.

Before each experimental session, the optimal intensity of stimulation evoking maximal compound action potentials (M_max_) and maximal twitch force was determined. Then, participants performed a standardized warm-up consisting of nine submaximal KE contractions (3 at 25%, 3 at 50% and 3 at 90% of their estimated maximum force), each lasting 5 s. Each experimental session included in the following order: 1) two or three 5 s maximal voluntary contractions (MVC) of the KE muscles; 2) a fatiguing exercise; 3) one MVC of the KE muscles, 4–5 s after the fatiguing exercise (Fig 1).

Each subject performed two MVCs of the KE muscles before the fatiguing exercise. A third MVC trial was performed if the difference in the peak force between the two MVC was greater than 5%. Each MVC effort lasted 5 s and a 4 min rest period was given between each MVC to avoid potential fatigue effects. Another MVC was performed 4–5 s after the fatiguing exercise. Standardized verbal encouragement was given during attempts to produce the maximal effort.

**Fig 2:**
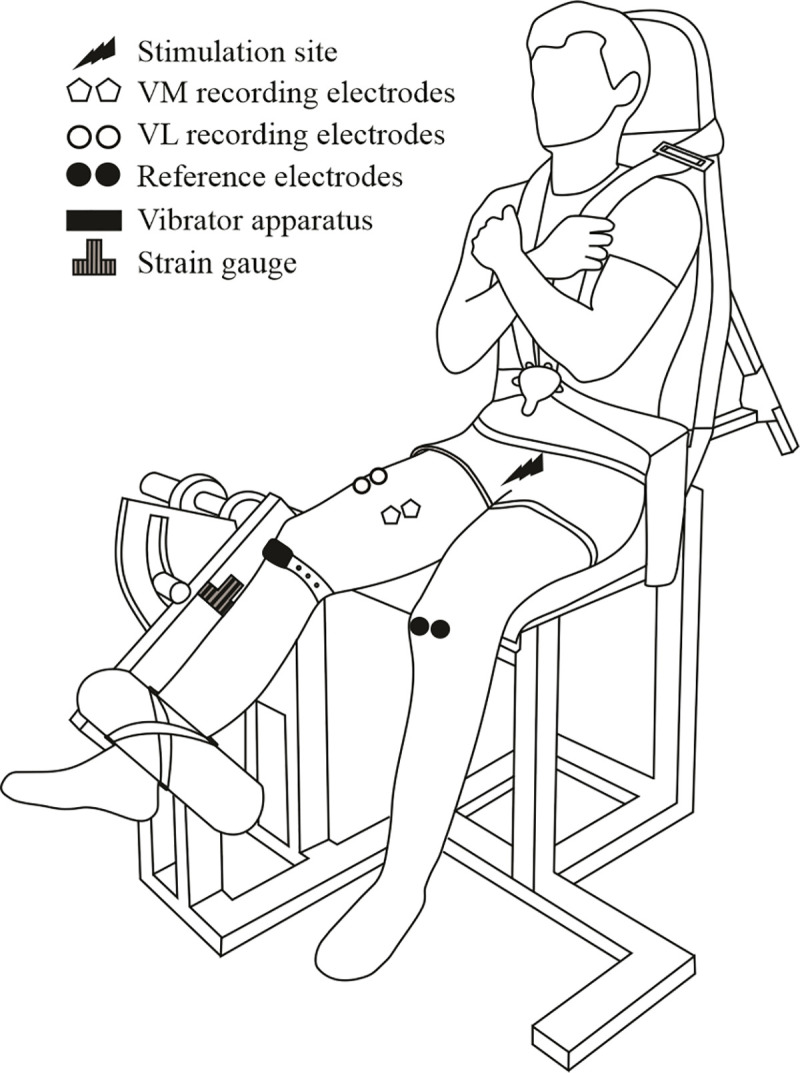
Illustration of experimental setup used during neuromuscular assessment and fatiguing exercise for the experimental session in which the individuals were tested at 150° knee angle. VL: Vastus Lateralis; VM: Vastus Medialis.

The fatiguing protocol consisted of three bouts of 10 min sustained KE contractions at an intensity corresponding to 10% MVC force. The force signal and a target line corresponding to 10% MVC force were displayed on a monitor in front of the subject to provide a visual feedback. Individuals were asked to maintain the force signal on the target line. A minute rest period was given between each bout. For the experimental session including vibration, low-amplitude mechanical vibrations (amplitude: 1 mm) were applied perpendicularly to the mid-portion of the right infrapatellar tendon using a commercial vibrator apparatus (Techno Concept, VB 115, Maine, France) during each of the three sustained contractions (Fig 2). The vibrator apparatus was strapped to the knee using elastic Velcro fasteners for the two protocols (i.e. vibration protocol and no-vibration protocol). To optimize the activation of Ia afferents, the vibration frequency was set at 80 Hz [29,30]. For the control session, the vibrator apparatus was placed over the infrapatellar tendon, but no vibration was applied, while participants performed the three submaximal KE contractions.

### Data collection

Mechanical responses associated with MVC and electrical stimulations of the KE muscles were collected using a home-made ergometer with a strain gauge (model CSSPE01345, Garos capteur, Bouguenais, France; calibration: 0 mV = 0 N; 3.5mV = -981 N France) (Fig 2). Participants were firmly attached to the ergometer by non-elastic belts, with a 90° hip joint angle (0° = full hip extension) and the right anatomical knee flexion-extension axis being aligned with the center of the ergometer’s rotation. The right leg was firmly attached with noncompliant straps to the ergometer’s accessory. In all of the experimental sessions, individuals were instructed to keep their head straight against the back of the chair while their arms crossed and grasped the two crossover shoulder belts, to avoid any movement (Fig 2).

Regarding electromyographic (EMG) recordings, the subject’s skin was carefully prepared by shaving, abrading and cleaning with alcohol. With respect to the SENIAM recommendation [31] and the underlying muscle fiber arrangement, two circular surface electrodes (Kendall Medi-Trace™, Canada) with a 1 mm diameter and an inter-electrode distance (center to center) of 2 cm were placed on the target muscles. For the vastus medialis (VM), a pair of electrodes was placed at 80% on the line between the anterior spina iliaca superior and the joint space in front of the anterior border of the medial ligament. For the vastus lateralis (VL), a second pair of electrodes was positioned at 2/3 on the line from the anterior spina iliaca superior to the lateral side of the patella. The reference electrodes were positioned on the patella of the left leg. The placement of the electrodes was marked on the skin with an indelible pen in order to ensure that the same recording site was used between the two experimental sessions (i.e. over 4 weeks). The EMG signal was collected using a BIOPAC MP35 system, sampled at 2 kHz, amplified (×500) and filtered with a band-pass filter 5 Hz–500 Hz. The EMG signals were stored with commercially available software (BIOPAC Student Lab Pro®, BIOPAC System Inc., Goleta, USA).

A high-voltage constant-current stimulator (maximal voltage 400 V, model DS7A, Digitimer, Hertfordshire, UK) was used to perform transcutaneous electrically-evoked contractions of the KE muscles. The stimulator was set to deliver rectangular pulses (1 ms) with a high voltage (400 V). The femoral nerve was stimulated using a surface electrode (Kendall Medi-Trace™, Canada) positioned over the nerve, in the femoral triangle. The anode was a rectangular electrode (50×90 mm; Stimex, Rouffach, France) located in the gluteal fold opposite to the cathode. The optimal intensity of stimulation required to evoke a M_max_ for VL and VM and maximal twitch force was determined at rest by progressively increasing stimulation intensity by 10 mA from 40 mA until the M_max_. The optimal intensity of stimulation was set to 120% of that required to elicit VL and VM M_max_ and maximal twitch force. Stimulation intensity was kept constant throughout the protocol (mean current, 84.4 ± 15.7 mA). A single electrical femoral nerve stimulation was delivered at rest 4 s before each MVC. A doublet (10 ms between 2 stimuli) was delivered over the isometric plateau (superimposed doublet) and 4 s after each MVC (potentiated doublet) to estimate the voluntary activation level (VAL) according to the interpolated twitch technique [32–34] (Fig 1).

### Data analysis

The MVC force was considered as the highest peak force value measured over two (or three) trials. Peak twitch force and peak doublet force were measured to assess peripheral fatigue [32,35]. The VAL was quantified by measurement of the superimposed force response to nerve stimulation during the MVC effort [36,37] to assess central fatigue. Because the superimposed stimulation is not consistently applied at the MVC peak force, the VAL was estimated according to the following formula, including the Strojnik and Komi [38] correction:

$$VAL=\left[1-\frac{superimposed\;doublet\;amplitude\times (T_{stim}/MVC\;force)}{potentiated\;doublet\;amplitude}\times 100\right]$$

VAL: maximal voluntary activation level; T_stim_: force value recorded at the time of the superimposed double stimulation; MVC force: maximal voluntary contraction peak force

Peak-to-peak amplitude of VL and VM M_max_ was measured from each single stimulus preceding the MVCs before and after the fatiguing exercises.

### Statistical analysis

The statistical analyses were performed using Statistica software 7.0 (StatSoft Inc., Tulsa, Oklahoma, USA). Data are presented as means ± standard deviation. A significance level of *P*<0.05 was used to identify statistical significance. All data being normally distributed (Shapiro-Wilk test), three-factor repeated measures ANOVAs [angle (90° and 150°) x condition (vibration and no-vibration) x time (before and immediately after fatiguing exercise)] were performed to compare each of the dependent variables (MVC force, VAL, peak twitch force, peak doublet force, and M_max_ for VL and VM muscles). When a main effect or a significant interaction was found, a post-hoc analysis was made using Tukey’s test. Partial eta squared ($\eta_{p}^{2}$) values have been reported as measures of the effect size, with $\eta_{p}^{2}$ > 0.14 considered to be a very large effect and 0.07 < $\eta_{p}^{2}$ < 0.14 considered to be a moderate effect [39].

## Results

All the mechanical and electrophysiological measurements are presented in Table 1.

**Table 1:** Mechanical and electrophysiological measurements according to angle, vibration condition and time.

	90°				150°					
	WITH VIBRATION		WITHOUT VIBRATION		WITH VIBRATION		WITHOUT VIBRATION		STATISTICS	
	MVC Pre	MVC Post	MVC Pre	MVC Post	MVC Pre	MVC Post	MVC Pre	MVC Post	Main effects	Interaction
**MVC force (N)**	1563±490	1228±338	1594±496	1274±384	1099±333	1006±344	1099±374	1008±407	*Angle-Fatigue*	Fatigue*Angle
**Single twitch force (N)**	315±80	269±103	310±145	252±112	147±52	140±59	145±69	120±60	*Angle-Fatigue*	*/*
**PD force (N)**	673±193	578±169	623±221	564±204	469 ±144	473±149	468±159	463±177	*Angle-Fatigue*	Fatigue*Angle
**VAL (%)**	93.6±4.1	87.5±5.6	95.8±3.0	91.6±6.7	90.9±10.4	90.2±12.0	91.8±9.7	89.9±10.6	*Fatigue*	Fatigue*Angle
**VL M**_**max**_ **(mV)**	6.4±4.6	5.9±3.8	6.3±4.2	6.0±4.2	7.0±2.9	6.6±3.0	6.5±2.7	6.2±3.0	*Fatigue*	*/*
**VM M**_**max**_ **(mV)**	9.8±4.0	9.2±3.9	8.2±3.5	7.3±3.2	8.9±3.2	8.9±3	9.0±3.6	8.8±3.7	*Fatigue*	Fatigue*Angle

Values are means ± SD; MVC: Maximal voluntary contraction; VAL: Voluntary activation level; PD: Potentiated doublet; Pre: before fatiguing exercise; Post: immediately after fatiguing exercise; VL: Vastus Lateralis; VM: Vastus Medialis; M_max_: maximal compound action potential.

### Maximal knee extensor force production

**Fig 3:**
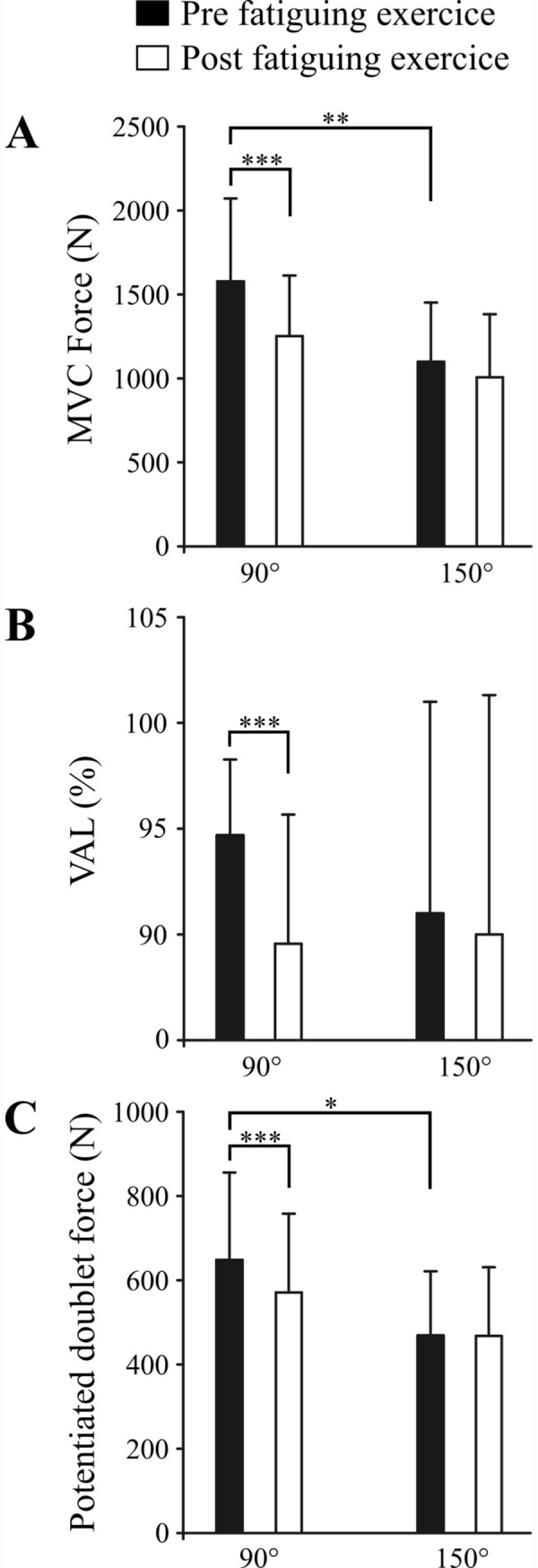
Effect of knee angle and fatiguing exercise on neuromuscular characteristics. *A* represents the effect of knee angle and fatiguing exercise on MVC force. *B* represents the effect of knee angle and fatiguing exercise on voluntary activation level, *C* represents the effect of knee angle and fatiguing exercise on potentiated doublet force. Note that this figure represents the significant interaction between angle and time, i.e. the conditions with vibration and without vibration are pooled. *MVC*: *Maximal voluntary contraction; VAL*: *Voluntary activation level*. * P <0.*05*, **P<0.*01*, ***P<0.*001*.

Statistical analysis performed for KE MVC force revealed a significant time effect (*P*<0.001; $\eta_{p}^{2}$ = 0.68) with an interaction angle × time (*P*<0.001; $\eta_{p}^{2}$ = 0.39) but no effect of the vibration condition was observed (*P* = 0.21; $\eta_{p}^{2}$ = 0.05). The post-hoc analysis showed that the KE MVC force was higher for the group^90°^ than the group^150°^ (*P*<0.05; $\eta_{p}^{2}$ = 0.18) before the fatiguing exercise (Fig 3A). The MVC force was significantly reduced (*P*<0.001) after the fatiguing exercise for the group^90°^. For the group^150°^, the MVC force was not significantly reduced but a strong tendency towards statistical significance was found (*P*<0.06).

### Twitch force and evoked potentials

Statistical analysis performed for peak twitch force revealed a significant angle (P<0.001; $\eta_{p}^{2}$ = 0.45) and time effect (P<0.01; $\eta_{p}^{2}$ = 0.28) but no effect of the vibration condition was observed (*P* = 0.21; $\eta_{p}^{2}$ = 0.04).

The ANOVA analysis performed for peak doublet force revealed a significant time effect (*P*<0.001; $\eta_{p}^{2}$ = 0.45) and an interaction angle × time (*P*<0.001; $\eta_{p}^{2}$ = 0.44) but no effect of the vibration condition (*P* = 0.22; $\eta_{p}^{2}$ = 0.04). The post hoc analysis showed that the peak doublet force was greater for the group^90°^ than that for the group^150°^ before the fatiguing exercise (648.3 ± 207.7 vs. 468.8 ± 152.1 N, respectively; *P*<0.001). Only the potentiated doublet force of the group^90°^ was significantly reduced after the fatiguing exercise (*P*<0.001).

Statistical analysis performed for M_max_ amplitude revealed a significant time effect of VL (*P*<0.05; $\eta_{p}^{2}$ = 0.13) and VM muscle (*P*<0.001; $\eta_{p}^{2}$ = 0.22) and an interaction angle × time of VM muscle (*P*<0.05; $\eta_{p}^{2}$ = 0.13). The post hoc analysis showed that the M_max_ was significantly reduced after the fatiguing exercise only for the group^90°^ (*P*<0.01).

### Voluntary activation level

The ANOVA analysis revealed a significant time effect (*P*<0.001; $\eta_{p}^{2}$ = 0.31) and an interaction between angle × time (*P*<0.05; $\eta_{p}^{2}$ = 0.13) for VAL but no effect of the vibration condition was observed (*P*>0.05; $\eta_{p}^{2}$ = 0.08). The post hoc analysis showed that the VAL was not different for the two knee angles (*P* = 0.61) before the fatiguing exercise but it was reduced after the fatiguing exercise only for the group^90°^ (*P*<0.001).

## Discussion

The aim of this study was to investigate the acute effects of an exercise, combining prolonged tendon vibration and weak submaximal isometric contraction, on maximal force production at two different knee angles. Significant neuromuscular fatigue of KE was observed for 90° knee angle but not for 150° knee angle. In contrast to our hypothesis, the neuromuscular fatigue, as revealed by the reduction in KE MVC force, observed at 90° knee angle was only the result of prolonged submaximal contraction. The tendon vibration did not therefore exacerbate the reduction in KE MVC force.

### Effect of tendon vibration

In our study, we investigated the effects of a single session of an exercise combining tendon vibration and submaximal fatiguing contractions on the maximal force produced by the quadriceps muscle. Because muscle length influences the effect of tendon vibration on neuromuscular function [40], we also investigated whether knee angle may change the acute effect of the combined exercise. No significant change in MVC force was found at the 150° knee angle during the fatigue-inducing protocols, with and without tendon vibration. In contrast, a significant neuromuscular fatigue was observed when the experimental protocol was performed at a 90° knee angle, without any differences between the vibration and control condition. This indicates that the fatigue-related combined exercise at a 90° knee angle was only the result of submaximal contractions but not of the tendon vibration. The lack of effect of muscle/tendon vibration on neuromuscular performance is in agreement with some reports for ankle plantar flexor [14,16], ankle dorsiflexor [15] and KE muscles [13]. However, this remains in contrast with other works in which it was observed that prolonged muscle/tendon vibration altered the maximal motor performance of several muscle groups, such as KE muscles [1,2,4,5,7–10]. These conflicting results might be also explained by the variability of experimental conditions among these studies such as subject’s morphological characteristics, vibration duration, frequency and amplitude, and the site of vibration application, i.e., muscle *vs*. tendon. In addition, it should be acknowledged that the present study did not assess the effects of tendon vibration on the Ia afferent activity, which according to our hypothesis could alter central fatigue. We also did not assess H-reflex excitability, which is known to decrease as a function of Ia afferent activity enhancement. Previous studies reported a decrease in H-reflex excitability without changes in MVC force after prolonged vibration at rest [14,15]. Therefore, it is possible that the vibration we applied in our participants altered the spinal loop, but this alteration was not sufficient to result in a greater decrement in MVC (or evoked) force and/or voluntary activation level compared to the control condition.

### Effect of submaximal contraction

Neuromuscular fatigue is defined as the decline in muscle performance associated with repeated muscle activity [41,42]. Sustained submaximal isometric contraction, even for low levels of intensity, can induce substantial neuromuscular fatigue [43–46]. For instance, it has been reported a decrease in KE muscle performances of ~27% [45] and ~28% [46] after prolonged submaximal contraction at 20% MVC until exhaustion. In accordance with these studies, our results revealed a large fatigue-related decrease in KE MVC force at 90° knee angles (-20.6%) but no significant fatigue for 150° knee angle, even if a strong decreasing trend was observed (-8.3%). Neuromuscular fatigue has been shown to be task dependent [41,45,47,48] and can be related to joint angle or muscle length [49]. This difference in muscle length may explain, therefore, the greater neuromuscular fatigue observed after submaximal isometric knee extensions performed at long muscle length (90° knee angle) compared to short muscle length (150° knee angle) [45,50–52]. It has been reported that an increase in actin-myosin crossbridges [52], a greater metabolic cost [51], a disruption of blood circulation [50] and an improved sensitivity of the myofilament to Ca^2+^ [53] for long muscle length compared to short muscle length, could explain the greater reduction in force production in the former condition.

In the present study, the greater reduction in MVC force at the 90° knee angle is partly explained by a greater alteration of the muscular contractility for the long muscle length compared to the short one. Indeed, we observed a peak twitch and doublet force reduction after the fatiguing exercise at the 90° knee angle but not at the 150° knee angle. This indicates that decreased excitation-contraction coupling only occurred after the fatiguing contractions performed at the 90° knee angle. This observation was corroborated by the VM M_max_, which was also significantly reduced at the 90° knee angle but not for the 150° knee angle. However, VL M_max_ is similarly reduced, for both the 90° knee angle and 150° knee angle, after the fatiguing exercises. It is worth noting that the decrease in M_max_ amplitude after the fatiguing exercise may also be explained by an alteration of the neuromuscular propagation [45,47,54]. This weak presence of peripheral fatigue at 150° could be explained by exercise that is not long enough and/or intensive enough to be able to generate an accumulation of metabolites [35,55].

The level of activation can also explain this observed force reduction. Before the fatiguing exercise, the activation level was lower at a short length compared to a long length, as previously shown [56–58]. A lower activation level corresponds to a reduced number and/or firing frequency of activated motor units. After the fatiguing exercise, the maximal VAL loss was greater for the group^90°^, suggesting a greater development of central fatigue during the sustained contraction at long compared to short KE length. This difference in fatigue could then be explained by a greater turnover of the motor units at 150°, allowing certain motor units to rest temporarily. Conversely, higher activation at longer lengths would reduce the possibility of motor unit rotation during fatiguing contraction, leading to a higher incidence of central fatigue for the same working time.

To conclude, this study demonstrated that a single session of infrapatellar tendon vibration and submaximal isometric contractions of KE induced a greater neuromuscular fatigue at the 90° knee angle compared to the 150° knee angle. However, this neuromuscular fatigue only resulted from voluntary submaximal contraction and is not exacerbated by tendon vibration.
